# Modulation of Alpha-synuclein Expression and Associated Effects by MicroRNA Let-7 in Transgenic *C. elegans*

**DOI:** 10.3389/fnmol.2017.00328

**Published:** 2017-10-13

**Authors:** Shamsuzzama, Lalit Kumar, Aamir Nazir

**Affiliations:** Laboratory of Functional Genomics and Molecular Toxicology, Division of Toxicology, CSIR-Central Drug Research Institute, Lucknow, India

**Keywords:** let-7, microRNA, *C. elegans*, Parkinson’s disease, RNAi

## Abstract

Neurodegenerative Parkinson’s disease (PD) is a multi-factorial disorder lacking complete cure. Understanding the complete mechanism of initiation and progression of this disease has been quite challenging; however, progress has been made toward deciphering certain genetic aspects related to the disease condition. Genetics studies have provided clues toward the role of microRNAs (miRNAs) in various disease conditions. One of the crucial miRNA molecules, let-7, is highly conserved miRNA and is known to regulate important functions of development and viability; its altered expression has been reported in *C. elegans* model of PD. We carried out studies with let-7, employing transgenic *C. elegans* model expressing ‘human’ alpha-synuclein and developed a let-7 loss-of-function model toward studying the downstream effects related to PD. We observed that let-7 miRNA was upregulated in *C. elegans* model of PD and figured that loss of let-7 miRNA leads to decreased alpha-synuclein expression, increased autophagy, increased Daf-16 expression, increased oxidative stress and increased lipid content with no effect on dopaminergic/acetylcholinergic neurons. Our findings indicate that let-7 miRNA regulates PD-associated pathways. Our study provides insight toward the role of let-7 in regulating expression of genes associated with these pathways which might have implications on the multi-factorial nature of PD. Potential pharmacological agents modulating the expression of let-7 could be studied toward targeting the multi-factorial aspect of PD.

## Introduction

Neurodegenerative Parkinson’s disease (PD) is an age-related disorder, and is characterized by the accumulation of Lewy bodies and Lewy neurites in substantia nigra pars compacta region of the brain. Lewy bodies are composed of alpha-synuclein protein in high proportion (Chaudhuri et al., 2015). PD affects 1–2% of the population and is the second most common among all neurodegenerative diseases (NDs). The symptoms of this disease include bradykinesia, muscle rigidity, cognition defects, tremors, as well as personality and behavioral disorders (Wong and Nass, 2012). PD is an incurable, multi-factorial disease which is associated with aggregation of misfolded proteins, alteration in levels of neurotransmitter dopamine, increase in neuronal cell death and disturbance in the ubiquitin–proteasome system (Gao and Hong, 2011). PD may result from genetic mutations, environmental exposure to toxins and is most commonly associated with old age. PD occurs most commonly in sporadic form rather than familial form. The familial form of PD is caused by the mutation in any of the proteins α-synuclein, perkin, *PINK1, UCHL1, DJ1*, or *LRRK2* genes. The familial form accounts only for 5–10% of patients (Douglas et al., 2007; Shulman et al., 2011) whereas, rest of the cases are sporadic in nature. No single or independent factor is known that could be targeted for combating PD, and for its multifactorial nature, the disease lacks a complete cure. It could be possible that studying miRNA molecules may help identify targets that may be helpful in treating multiple pathways that go awry in PD patients.

MicroRNAs are endogenous evolutionarily conserved 20–25 nucleotide long non-coding RNAs. These molecules function via being trans-acting factors and regulate gene expression machinery at post-transcriptional level. miRNAs inhibit protein synthesis either by degradation of targeted mRNA or by inhibition of its translation. Some recent studies report that miRNAs regulate gene expression at transcriptional level (Selbach et al., 2008; Bicchi et al., 2013). Alterations in the functions/biogenesis of miRNAs have been linked to multiple ailments that include neurodegenerative diseases (NDs), cancer, cardiovascular disease, and diabetes mellitus (Sonntag, 2010; Kumar et al., 2012; Dimmeler and Nicotera, 2013; Kocerha et al., 2014). Like most coding genes, miRNA genes are transcribed through RNA polymerase II transcriptional activity, generating hairpin like structure called primary transcript. Within nucleus these primary transcript pri-miRNAs, are processed by microprocessor complex protein resulting in precursor miRNAs (pre-miRNAs). After that, pre-miRNAs are transported into cytoplasm via expotin-5 from nucleus. Cytoplasmic pre-miRNAs are further processed to generate mature miRNAs which are incorporated into RNA-induced silencing complex followed by inhibition of target mRNA either by degradation of mRNA or by repression of translation (Ling et al., 2013; Chaudhuri et al., 2016; Shamsuzzama et al., 2016). miRBase21 database^1^ illustrates the presence of 434 predicted mature miRNAs in *C. elegans*, and 2,588 mature miRNAs in *Homo sapiens*, although an actual number of miRNAs may be higher.

In mammals, miRNAs play important role in the development of brain, neuronal specification, function, and maintenance (Krichevsky et al., 2003, 2006; Sempere et al., 2004; Schratt et al., 2006). Most of the specific genes required for cellular identity are regulated by miRNAs thus suggesting that miRNAs may have significant role in the development of complex tissue and organs of higher organisms (Lee et al., 2006; Lu et al., 2007).

Let-7 miRNA is 22 nt long non-coding RNA, which was first discovered in *C. elegans*. It is highly conserved across animal species and the let-7 family consists of 9, 14, and 13 members in *C. elegans*, mouse and humans, respectively (Shamsuzzama et al., 2016). Let-7 miRNA is found to be downregulated in different types of cancer including lung cancer, breast cancer, colon cancer, gastric cancer, and Burkitt’s lymphoma. Let-7 miRNA acts as tumor suppressing miRNA and may well come up as an interesting target for various cancers (Barh et al., 2010). Let-7 directly regulates oncogenic genes that are involved in signaling pathways in tumor progression. Oncogenes that are regulated by let-7 are *ras, hgma2, myc, NIRF* and JAK-STAT3 pathway molecules (Wang et al., 2012). There is very little that is known about the role of let-7 miRNA in the progression of PD. However, some studies have shown that its expression levels were altered in *C. elegans* model of PD (Asikainen et al., 2010), which implies that let-7 miRNA networking pathways may be playing a critical role in PD development.

In order to investigate the role of let-7 miRNA in PD and its associated factors we designed RNAi feeding bacterial clone of let-7 miRNA toward knocking down let-7 miRNA in the nematodes and studied its effect on disease model for various endpoints, including investigation of alpha-synuclein protein expression, lipid content, oxidative stress, quantification of autophagy/apoptosis marker genes, dopaminergic neurodegeneration and associated phenotypes. We found that loss of let-7 miRNA leads to decreased alpha-synuclein expression, increased autophagy, increased Daf-16 expression, increased oxidative stress and increased fat content with no effect on dopaminergic/acetylcholinergic neurons. Our study provides understanding of the role of miRNA let-7 in PD and confirms that absence of let-7 miRNA leads to decrease in accumulation of alpha-synuclein protein in transgenic *C. elegans*. Our studies further provide evidence that let-7 possibly decreases alpha-synuclein expression via increasing autophagy and increasing *daf-16* forkhead box O (FOXO) transcription factor.

## Materials and Methods

### *C. elegans* Culture and Maintenance

*C. elegans* strains were cultured using standard techniques as described previously (Brenner, 1974). *Escherichia coli* (*E. coli*) strain-OP50 (uracil auxotroph) was used as standard food. To obtain age synchronized animals, procedure described previously was followed (Stiernagle, 2006). In brief, worms were washed with M9 buffer and then treated with axenizing solution (5 mL of 1M sodium hydroxide solution and 2 mL of sodium hypochlorite) until the eggs were released from the dissolved worm bodies.

### *C. elegans* Strains

Strains employed in this study were N2, wild-type Bristol; NL5901, pkIs2386 [unc-54p::alpha-synuclein::YFP + unc-119(+)]; BZ555, egIs1 [dat-1p::GFP]; LX929, vsIs48[unc-17::GFP]; DA2123, adIs2122 [lgg-1p::GFP+ rol-6(su1006)]. All the strains were procured from the *Caenorhabditis* Genetics Center (University of Minnesota).

### Genomic DNA Isolation

Genomic DNA from mixed population of *C. elegans* (N2 Bristol) was isolated using PureLink^®^ Genomic DNA Kit (Invitrogen, cat no. K1820-01) as described in the manufacturer’s manual. Briefly, worms were washed with M9 buffer, lysed by adding 180 μl pure link genomic digestion buffer, 20 μl proteinase K and kept at 55°C in water bath for 3 h. Afterward, 200 μl pure link genomic lysis/binding buffer, 200 μl 100% ethanol were added, mixed well and transferred to pure link spin column. This was followed by centrifugation at 10,000 × *g* for 1 min at RT. Column was washed with provided wash buffers and eluted with pure link genomic elution buffer.

### Plasmid Constructs

Plasmids were constructed using standard techniques as described previously (Fraser et al., 2000). In brief let-7 miRNA gene sequence was retrieved from WormBase (sequence number C05G5.6). The 99 bp full length was amplified using standard PCR with a set of primers having sacI and kpnI restriction sites. A 25 μl reaction mixture containing 50 ng of *C. elegans* genomic DNA, 400 nanomolar forward and reverse primer, 10 mM dNTPs was prepared followed by incubation at 95°C for 10 min (1 cycle), 94°C for 30 s, 55°C for 30 s, 72°C for 30 s (30 cycle), and 72°C for 5 min (1 cycle). Amplified product was subcloned in TA (pCR^®^ 2.1 vector) (Invitrogen cat no. 450046) and then cloned employing Timmons and Fire feeding vector L4440 (Addgene plasmid 1654), and transformed into HT115 (DE3), an RNase III-deficient *E. coli* strain with IPTG inducible T7 polymerase activity. Colonies containing correct sized insert were confirmed with double digestion by sacI and kpnI restriction digestion.

Forward primer – GAG CTC TAC ACT GTG GAT CCG GTG AGG T (Tm- 59.2)

Reverse primer – GGT ACC TCG AAG AGT TCT GTC TCC GGT A (Tm- 57.8).

let-7 sequence (C05G5.6);

tacactgtggatccggtgaggtagtaggttgtatagtttggaatattaccaccggtgaactatgcaattttctaccttaccggagacagaactcttcga.

### Isolation of Non-coding Small RNA Using mirVana^TM^ miRNA Isolation Kit

The extraction of non-coding was carried out employing standard procedure, briefly, water treated with 0.2% diethyl pyrocarbonate (DEPC-Sigma, Cat. No.-D5758) was used to remove adhering bacteria from age synchronized N2 and let-7 silenced groups. miRNA was isolated using mirVana^TM^ miRNA isolation kit (Ambion P/N AM1561) as per instruction provided within the manufacturer’s manual. Briefly, 250 μl lysis/binding buffer was added followed by homogenization and addition of 1/10 volume miRNA homogenate additive, the solution was mixed well and kept on ice for 10 min. After that equal amount of acid-phenol:chloroform was added to the lysate, it was mixed well and centrifuged at 10,000 × *g* for 5 min., the aqueous phase was removed, and 1.25 times volume of 100% ethanol was added followed by filtration via passing through filter cartridge, washing, and elution.

### Reverse Transcriptase Reaction

Isolated non-coding small RNAs were converted into cDNA using TaqMan^®^ MicroRNA Reverse Transcription kit (Applied Biosystem cat no. P/N 4366596). The 15 μl reactions were incubated in an Agilent sure Cycler 8800 for 30 min at 16°C, for 30 min at 42°C, 5 min at 85°C and then held at 4°C.

### TaqMan miRNA Assay

Quantification of miRNA was carried out using TaqMan^®^ Universal Master MixII (Applied Biosystem cat no. 4440040). The 20 μl maxima contain 1X TaqMan^®^ Universal Master MixII no UNG, 1X TaqMan^®^ assay and 100 ng cDNA template. The program for amplification was 95°C for 10 min (1 cycle), followed by 95°C for 15 s and 60°C for 1 min (40 cycles). Experiment of each sample was carried out in triplicate sets. Fold change of all samples were analyzed using comparative 2^-ΔΔCT^. U18 was used as endogenous control for normalization of miRNAs expression.

### Prediction of Targets and Pathway Analysis of Let-7 miRNA Molecules

We used DIANA TOOLS – mirPath v.3 for target prediction and pathway analysis. DIANA TOOLS works based on specifically designed algorithms and database toward mining available information pertaining to test molecules and associated pathways. Enrichment analysis of target genes of miRNA to Kyoto Encyclopedia of Genes and Genomes (KEGG) pathways is performed by this software thus providing with an overview of pathway which might be regulated by specified miRNAs.

### Real-time PCR (qPCR) Assay

Age synchronized control and let-7 knockdown worms were washed twice with 0.2% DEPC (Sigma, Cat. No.-D5758) treated water to remove adhering bacteria, following which total RNA was isolated using RNAzol^®^ RT method (Sigma, Cat. No. R4533), and quantified through NanoDrop (Thermo, Quawell, UV-Vis Spectrophotometer, Q5000). About 5 μg of total RNA was used for the synthesis of cDNA using RevertAid First Strand cDNA synthesis kit (Thermo Scientific, Cat. No. #K1622). Quantification of mRNA level was carried out using SYBR^®^ Select Master Mix (Applied Biosystems cat. No.4472908) chemistry as described previously (Jadiya et al., 2012). In brief cDNA equivalent to 125 ng was amplified in 20 μl maxima using Agilent MX3005P-detection system (Agilent Technologies). The program for amplification was, 50°C for 2 min 95°C for 10 min (1 cycle), followed by 95°C for 30 s, 55°C for 30 s and 60°C for 30 s (40 cycle) and melting curve detection (95°C for 5 s, 65°C for 1 min). Experiment of each sample was carried out in duplicate sets. Fold change of all samples was analyzed using comparative 2^-ΔΔCT^. Integrated DNA Technologies (IDT) software was used for designing of primers of desired genes. *act-1* mRNA was used as endogenous control for normalization. Primers sequences of genes used are as follows:-

*act-1* forward: TTA CTC TTT CAC CAC CAC CGC TGA

*act-1* reverse: TCG TTT CCG ACG GTG ATG ACT TGT

*lgg-1* forward: AAC AAC TTT GAG AAG CGT CGT GCC

*lgg-1* reverse: TCT TCT GGA CGA AGT TGG ATG CGT

*atg-5* forward: TGA TGA AAG ACG AGT CGG CAC AGT

*atg-5* reverse: GTT TGG CAG TGA TTA GGG CCT GTT

*atg-7* forward: CGC TTG GAT GTA ACA TTG CCC GTT

*atg-7* reverse: AAT GCG TTG GAT AGC AGC TTG TGC

*atg-13* forward: ACT CCA GAA GAC AAA GAG CCA ACG

*atg-13* reverse: TTG GCG CAC CAC CGA AAT CTG ATA

*vps-34* forward: TGG ATC CCT TTG CAT CAC GAC GTA

*vps-34* reverse: CGA AAC AAT CCC AAC ACC ACC GTT

*bec-1* forward: AGG AGC TGG AGC AAC AGT TGA AGA

*bec-1* reverse: ATA TTG ACG TTC GGC TTC CAG CGA

*ced-4* forward: AGT GCT CTT GCT TTC GCA GTT GTG

*ced-4* reverse: TGA GAA GAG CTC CAC GTT TGC TGA

*cep-1* forward: AGT CGT CTT CAT GGA TGC GTT CCT

*cep-1* reverse: TTG CGT CGG AAC CCA AGT GTA TCT

*lin-45* forward: AGT CTG CCG AGA TGT GCT TCT TCA

*lin-45* reverse: TTG TCA CTT GTT CCT GCT CCT CCA

*nsy-1* forward: TCT TCA TTC CAC GTT GTG CCA TGC

*nsy-1* reverse: ACC CTC CAG AAT TTG CTT CCC GTA

*jnk-1* forward: TGG CTG GTT CCA TCA TCA TCT GGT

*jnk-1* reverse: CGT TTG AGA ACA ACC ATC TGC GCT

*jkk-1* forward: GAA GCT GCT GCG TCG CAT TTA TCA

*jkk-1* reverse: ACA CAG CTT TAC ATT GCC GCT GTC

*daf-16* forward: GCG AAT CGG TTC CAG CAA TTC CAA

*daf-16* reverse: ACA CGA TCC ACG GAC ACT GTT CAA

*daf-12* forward: GCT CCT GGT ATG AAT GGG TATC

*daf-12* reverse: ACT CTC TTC GCT GGA GTC TAA

### Silencing of Let-7 miRNA

The knockdown of let-7 miRNA was achieved by employing standard feeding protocol as described previously (Fraser et al., 2000). dsRNA expressing bacterial clone targeted for *C. elegans* miRNA, was cultured for 6–8 h in LB containing 50 μg/ml ampicillin, then seeded onto NGM plates with 5 mM IPTG and 25 mg/L carbenicillin followed by an overnight incubation at 22°C to induce expression. Synchronous populations of embryos were transferred onto these plates and control (EV) for further studies.

### Assay for Alpha-synuclein Protein Accumulation

Expression of alpha-synuclein protein was examined in control and let-7 miRNA knockdown worms of the NL5901 strains as described previously (Jadiya et al., 2012). In brief after 48 h of treatment, worms were washed with M9 buffer up to three or four times until all adhering bacteria were removed. Worms were immobilized with 100 mM sodium azide (Sigma, cat no. 71289) and placed onto slides with agar pads (2% agarose) followed by sealing with cover slip. Imaging of immobilized worms was carried out using fluorescence microscope (Carl Zeiss) to monitor alpha-synuclein protein expression. In each group minimum 10 images were taken for analysis and each experiment was repeated thrice. ImageJ software (ImageJ, National Institutes of Health, Bethesda, MD, United States) was used to quantify the expression of protein by measuring fluorescence intensity.

### Assay for Autophagy in Worms

Transgenic strain DA2123 (LGG-1::GFP) was used for assessment of autophagy. LGG-1 is associated with autophagosomal membrane and has been widely used for autophagy detection (Jenzer et al., 2014; Manil-Segalen et al., 2014). LGG-1 is the ortholog of the mammalian LC3 and *Saccharomyces cerevisiae* Atg8 protein. In this study, control and let-7 miRNA silenced worms were washed with M9 buffer to remove any adhering bacteria. Worms were immobilized with 100 mM sodium azide (Sigma, cat no. 71289) onto 2% agar padded slides and sealed with a cover slip. Imaging of live (immobilized) worms was done using fluorescence microscope (Carl Zeiss) at 63× oil magnification to monitor punctate GFP. Experiments were repeated three times and five worms were taken for analysis for each individual group.

### Assay for Motility of Worms

Motility defect due to neurotransmitter imbalance is one of symptoms of PD. Motility of worms was quantified through thrashing assay in which number of thrashes was counted as per previously described method (Buckingham and Sattelle, 2009). One thrash was defined as complete bending of the body one way to the outermost angle and back to the initial posture. In this study, worms from different assay conditions were washed with M9 buffer to eliminate adhering bacteria; a single worm was placed on a drop of M9 buffer. The timer was set at 30 s and sigmoidal body bends were counted under stereo-zoom microscope (Leica). Ten worms were counted for every group; experiments were repeated thrice.

### Estimation of Reactive Oxygen Species (ROS)

In order to explore the effect of let-7 miRNA on the level of oxidative stress, we carried out estimation of ROS levels using 2′,7′-dichlorodihydrofluorescein diacetate (H_2_DCFDA) assay following the protocol as described by Kaur et al. (2012). Worms of control and let-7 miRNA silenced groups were washed thrice with M9 buffer and twice with phosphate buffer saline (PBS). An approximate number of 100 worms/100 μl assay solution, were transferred to assay wells of OptiPlate-96 F (Perkin Elmer) with each group being assayed in triplicates. A volume of 100 μl H_2_DCFDA (Cat. No. D399, Invitrogen) from an ethanol stock of 100 μM, was added to each well. Fluorescence from each well was quantified at three time points – (i) before addition of the dye, (ii) immediately after addition of the dye, and (iii) post 1 h incubation of addition of the dye. The Fluorescence intensity measurements were carried out using Multimode plate reader (Perkin Elmer, VICTOR^TM^ X3), at excitation wavelength 485 nm and an emission wavelength 520 nm. The change in fluorescence was calculated by subtracting initial reading from the final reading; the numbers were presented as fluorescence intensity per worm and plotted as mean ± SE. Statistical significance was calculated by student’s *t*-test using GraphPad Prism software package.

### Assay for Fat Content in Nematodes

The effect of let-7 miRNA silencing on fat content in nematodes was studied by staining worms with Nile red (MP Biomedicals cat no. 151744) a fat staining dye. Nile red was mixed with control (EV)/let-7 miRNA RNAi clone and seeded onto NGM-IPTG plates followed the protocol as described previously (Ashrafi et al., 2003). Synchronous aged embryos derived from sodium hypochlorite treatment were transferred onto Nile red pre-mixed plates and kept for 48 h at 22°C. After 48 h, synchronized worms were washed off with M9 buffer two to three times to eliminate adhering bacteria. Worms were mounted with 100 mM sodium azide (Sigma, cat no. 71289) using agar padded cover slip on a glass slide. The extent of fat content was analyzed using fluorescence microscope (Carl Zeiss). Fluorescence intensity was quantified using ImageJ software (ImageJ, National Institutes of Health, Bethesda, MD, United States). Five worms were analyzed for each group and experiments were repeated thrice.

### Studies on Acetylcholinergic and Dopaminergic Neurons

Here, we employed transgenic *C. elegans* strain LX929 (*unc-17*::GFP; expressing GFP under the influence of the *unc-17* promoter specifically in cholinergic neurons) and BZ555 (P*dat-1*::GFP; expressing GFP under the influence of the *dat-1* promoter specifically in the dopaminergic neurons) for assaying the effect of let-7 miRNA silencing on acetylcholinergic and dopaminergic neurons (Pu and Le, 2008; Barbagallo et al., 2010). Worms of different groups were washed with M9 buffer to remove adhering bacteria. The 100 mM sodium azide (Sigma, cat no. 71289) was used for anesthetizing the worms and mounted onto glass slide with agar padded cover slip. Fluorescence intensity measurement for GFP was carried out using fluorescence microscope (Carl Zeiss). Experiments were repeated thrice and imaging was carried out for a minimum 10 worms in each individual group.

### Effect on Dopamine Associated Function

Dopamine is a neurotransmitter of phenethylamine and catecholamine families. It plays important role in the regulation of motor behavior of *C. elegans* and also plays important role in functions like olfaction. Response of *C. elegans* to volatile attractants and repellents depends upon the levels and functioning of dopamine. Any alteration in dopamine leads to defects in motor function in response to foods and repellents (Sharples et al., 2014). We studied effect of dopamine in worms employing well established assay, i.e., nonanol repulsion assay (Jadiya et al., 2016). A single worm was placed in NGM plate and a drop of 1-nonanol was put near the head of the worm. The time taken by the worm in responding to the repellent by its ‘repulsion response’ was assayed for 10 worms of each group. The mean time of repulsion (in seconds) was calculated and the statistical significance of test groups was calculated with reference to control.

### Statistical Analysis

Statistical analysis was carried out using GraphPad software package; statistical significance of data was calculated employing student’s *t*-test. All data are presented as mean ± standard error of the mean.

## Results

### Let-7 miRNA Was Over-expressed in *C. elegans* Model of PD

**FIGURE 1:**
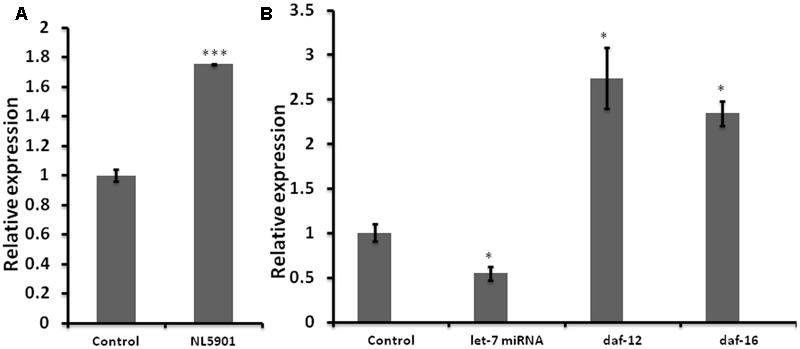
Graph depicting relative expression of let-7 miRNA and its targets, studied through real-time PCR (qPCR). **(A)** Expression level of let-7 miRNA in PD model vs. wild type. **(B)** Expression level of let-7 miRNA and their targets in let-7 knockdown worms. ^∗^*p* < 0.05, ^∗∗∗^*p* < 0.001.

Impaired miRNA expression is known to be associated with the development and progression of neurodegenerative PD (Wong and Nass, 2012). We employed *C. elegans* model of PD, (NL5901) for quantification of the expression level of let-7 miRNA. NL5901 is the transgenic strain expressing wild type “human” alpha-synuclein protein in body wall muscle under the control of unc-54 promoter. This strain is designed in such a way that the alpha-synuclein expression is driven to muscle cells via unc-54 promoter. Considering the fact that neuronal cells are largely refractory to RNAi mediated silencing, studying such effects in muscles makes it effective and the YFP expression can be detected via microscopy. Transgenic strains expressing either wild type or mutant alpha-synuclein protein under pan neuronal promoter have shown similar effect on locomotion (Lakso et al., 2003). In order to quantify the expression level of let-7 miRNA, we carried out TaqMan based real-time PCR studies for let-7 miRNA in wild type (N2) and alpha-synuclein expressing strain (NL5901) of *C. elegans*. We observed that let-7 miRNA was overexpressed in PD model by 75% (*p* < 0.001) as compared to that of control group (**Figure 1A**). This, intriguingly, is in contrast to the findings gathered studying ‘mutant’ alpha-synuclein, as against wild type species studied by us. Asikainen et al. (2010) reported that let-7 miRNA was downregulated in transgenic strain expressing mutant alpha-synuclein (A53T). This opposite effect could be attributed to the fact that the previous studies reported data on “mutant’ alpha-synuclein species whereas our studies were conducted on the ‘wild type’ alpha-synuclein.

### Mature Let-7 miRNAs Were Downregulated in Let-7 miRNA Silenced Worms

Reverse genetics is a widely used method in functional genomics research. Knockdown of genes via RNA interference has been well established and widely accepted tool for studying the function of target genes in *C. elegans*. Keeping this in mind we created RNAi feeding bacterial clone for let-7 miRNA in order to decipher its function. For the validation of RNAi mediated inhibition we carried out TaqMan miRNA assay toward quantification of let-7 miRNA levels under untreated and let-7 miRNA silenced conditions. We observed that let-7 miRNA was reduced by 46% (*p* < 0.05) in let-7 miRNA silenced worms as shown in **Figure 1B**.

### RNAi of Let-7 miRNA Resulted in Upregulation of Downstream Target Genes

miRNAs are endogenous short nucleotide targets of mRNA which suppress target gene function either by its degradation or repression of translation. Single mature miRNA could target hundreds of mRNA molecules at the same time. We carried out quantitative real-time PCR studies toward quantification of the mRNA levels of *daf-12* and *daf-16* in worms of control and let-7 miRNA silenced groups. DAF-12 is a nuclear hormone receptor; a member of steroid hormone receptor superfamily. *daf-12* mRNA is negatively regulated by let-7 miRNA. DAF-12 affects dauer formation and aging via TGF and insulin signaling pathway (Grosshans et al., 2005). DAF-16 homolog of mammalian FOXO transcription factor plays important role in neuroprotection (Tehrani et al., 2014). We observed that there was a significant 174% (*p* < 0.05), and 134% (*p* < 0.05) upregulation of *daf-12* and *daf-16*, respectively, as compared to control (**Figure 1B**) that validated the role of let-7 miRNA in the regulation of *daf-12 (*previously reported; Grosshans et al., 2005) and predicted *daf-16* genes (miRBase21)^2^.

### Pathway Analysis of Let-7 miRNA

**FIGURE 2:**
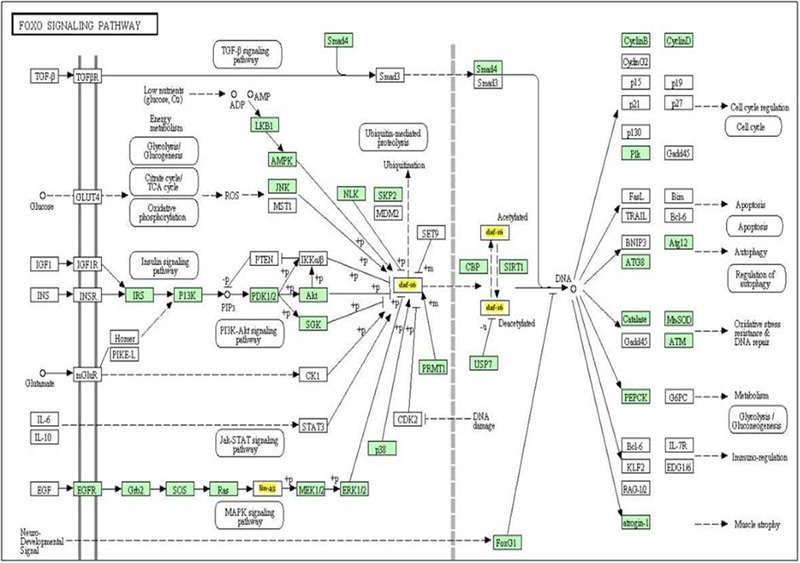
The KEGG pathway “FOXO signaling pathway” is regulated by let-7 miRNA. Target genes of let-7 miRNA are denoted by yellow color.

It is well known that single miRNA molecule might regulate multiple genes simultaneously (Qiu et al., 2011) thereby regulating multiple pathways. In order to investigate which biological pathways are affected by let-7 miRNA, we applied miRPath v.3, a miRNA pathway analysis tool. According to findings gathered from this tool, let-7 miRNA is involved in pathways of apoptosis, autophagy, cell cycle regulation, glycolysis/gluconeogenesis, MAPK signaling pathway and P13K-Akt signaling pathway (**Figure 2**).

### Knockdown of Let-7 miRNA Led to Reduced Expression of Alpha-synuclein Protein

**FIGURE 3:**
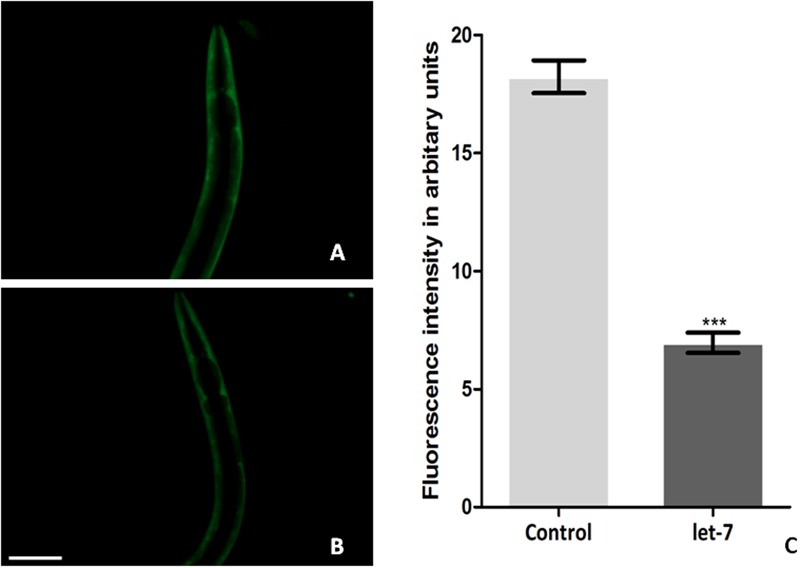
Alpha-synuclein expression in NL5901 strain of *C. elegans* (studied through fluorescence microscopy) fed on control **(A)** and let-7 miRNA knockdown condition **(B)**. **(C)** Graphical representation of fluorescence intensity as quantified using ImageJ software (^∗∗∗^*p* < 0.001). Scale bar: 50 μm.

Silencing let-7 in NL5901 transgenic strain led to decreased accumulation of alpha-synuclein. Accumulation of protein was deliberated as YFP expression pattern in the transgenic strain NL5901. Worms in the control group (NL5901 fed on EV) expressed optimal level of alpha-synuclein protein (**Figure 3A**), while let-7 miRNA silenced worms showed reduction in the level of alpha-synuclein protein (**Figure 3B**). The knockdown of let-7 miRNA decreased the fluorescence intensity of alpha-synuclein::YFP by 2.82-fold (*p* < 0.001) when compared to control worms; with mean fluorescence intensity for the control group 31.57 ± 0.5497 (*N* = 10) arbitrary units and that for let-7 miRNA knockdown worms was 11.18 ± 0.2047 (*N* = 10) arbitrary units (**Figure 3C**).

### Knockdown of Let-7 miRNA Influenced the Expression of Autophagy Marker Genes

**FIGURE 4:**
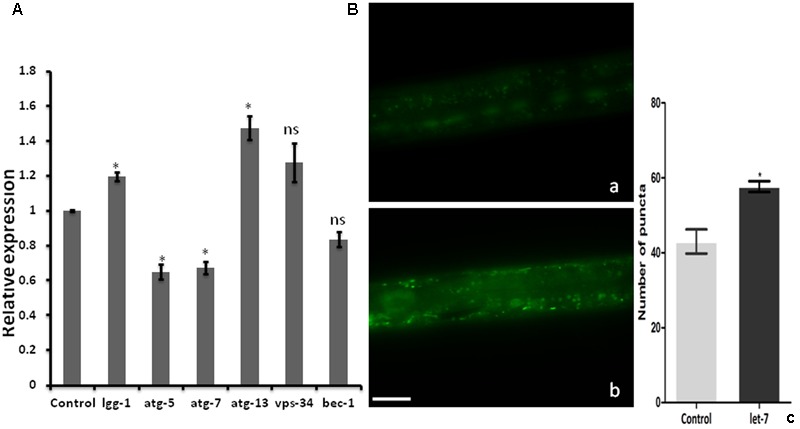
Assay for autophagy marker genes in *C. elegans*; **(A)** relative expression of autophagy marker genes studied through real-time PCR after let-7 miRNA silencing **(B)**: Expression pattern of LGG-1::GFP in DA2123 using fluorescence microscope; control (a), let-7 knockdown (b), number of puncta as quantified using ImageJ software (c). ^∗^*p* < 0.05, ns, not significant. Scale bar: 50 μm.

Clearance of damaged organelles and protein aggregates is associated with autophagy. Mutation in autophagy regulating genes is known to be related with NDs (Nixon, 2013). To explore the function of let-7 miRNA in autophagy mediated neuroprotection, we studied known autophagy marker genes (Yue et al., 2009) and quantified their mRNA levels using quantitative real-time PCR (qPCR) in normal and let-7 miRNA silenced condition. We found that expression level of lgg-1 and atg-13 mRNAs was significantly upregulated by 19% (*p* < 0.05) and 47% (*p* < 0.05) while mRNA levels of atg-5 and atg-7 were significantly downregulated by 35% (*p* < 0.05) and 33% (*p* < 0.05), respectively, when compared to that of worms from control group (**Figure 4A**). *lgg-1* is an ortholog of *Saccharomyces cerevisiae* Atg8p and mammalian MAP-LC3, which is required for degradation of cellular components, *atg-13* is an autophagy related gene required for autophagosome formation. The process of autophagy is known either to be dependent or independent of atg-5/atg-7 pathway (Nishida et al., 2009). We observed that knocking down of let-7 miRNA led to increase in lgg-1 and atg-13 whereas it led to decrease in atg-5 and atg-7 expression. These results suggest that absence of let-7 miRNA exerts its effects via atg-5/atg-7 independent alternative pathway for clearance of misfolded aggregated proteins.

### GFP::LGG-1 Was Increased in Let-7 miRNA Silenced Condition

As we observed a significant induction in expression of autophagy related genes, we went on to study the formation of autophagosomal vesicles employing a GFP::LGG-1 strain in which increased GFP puncta reflect an increase in autophagosome formation (Alberti et al., 2010). We observed an increase in punctae by 34.11% (^∗^*p* < 0.05) in let-7 miRNA silenced worms as compared to that control worms. The mean punctae for the let-7 miRNA silenced worms was 57.67 ± 1.453 (*N* = 5) whereas it was 43.00 ± 3.215 (*N* = 5) for the control group (**Figure 4B**).

### Expression of Apoptosis Marker Genes Was Altered in Let-7 miRNA Silenced Worms

**FIGURE 5:**
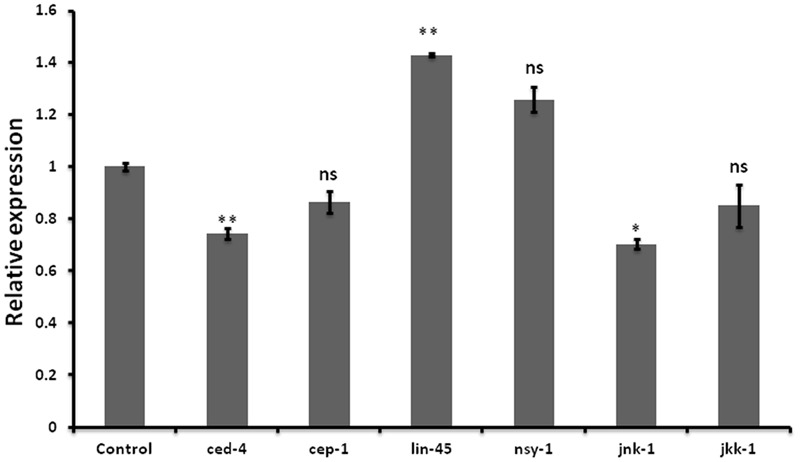
Graphical representation of relative *mRNA* expression of apoptosis pathway genes after let-7 miRNA silencing using qPCR analysis in *C. elegans.* ^∗∗^*p* < 0.01, ^∗^*p* < 0.05, ns, not significant.

Neurodegenerative diseases including Alzheimer’s disease, PD Amyotrophic lateral sclerosis, and Huntington disease are characterized by neuronal cell death (Mattson, 2000). Wishing to delineate the possible role of let-7 in multifactorial aspect of PD, we further examined the effect of let-7 miRNA knockdown on the expression level of genes associated with cell death. We carried out quantitative real-time PCR of some of previously reported genes of cell death (Cecconi et al., 1998; Kuan et al., 1999; Hayakawa et al., 2011; Rutkowski et al., 2011; Jiang and Wu, 2014) under control and let-7 miRNA silenced condition. We observed that expression levels of *ced-4* and *jnk-1* mRNA were significantly downregulated by 26% (*p* < 0.05) and 30% (*p* < 0.05), respectively, whereas mRNA level of lin-45 was upregulated by 42% (*p* < 0.05) as compared to that control group (**Figure 5**). *ced-4* mediates programmed cell death by activating *ced-3* (Aballay and Ausubel, 2001). *jnk-1* encodes a serine/threonine kinase required for apoptotic signaling in both extrinsic and intrinsic pathways (Dhanasekaran and Reddy, 2008). *lin-45* encodes an ortholog of vertebrate protein RAF which is required for vulval differentiation (Han et al., 1993). In our study, we found that let-7 miRNA silenced worms showed downregulation of *ced-4* and *jnk-1* while upregulation of lin-45 mRNA. Thus knocking down of let-7 miRNA protects cell death by reducing the expression level of *ced-4* and *jnk-1* as well as via maintaining vulval viability by increasing the expression level of *lin-45*.

### Let-7 miRNA Silenced Worms Exhibited Enhanced Motor Function in Transgenic Strain NL5901

**FIGURE 6:**
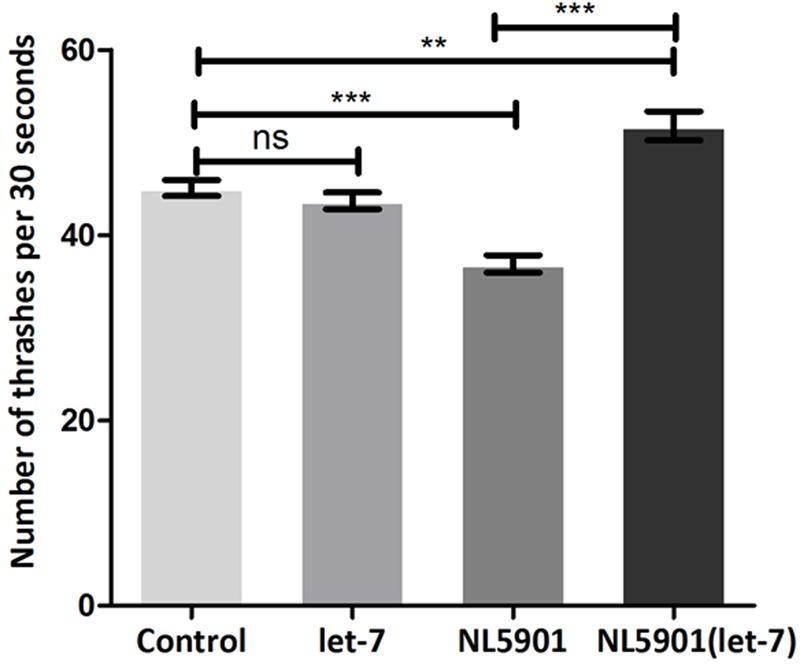
Graphical representation of thrashing pattern in wild type N2 strain and human alpha-synuclein expressing NL5901 strain. ^∗∗∗^*p* < 0.001, ^∗∗^*p* < 0.01, ns, non-significant.

Knockdown of let-7 miRNA exhibited no marked effect on motility in wild type strain N2. However, motility was significantly increased after knockdown of let-7 miRNA in NL5901 strain as compared to that of N2 and NL5901. We observed a mean thrashing count of 45.10 ± 0.8622 (*N* = 10) and 36.90 ± 0.9481 (*N* = 10) in the worms of control group N2 and NL5901, respectively. Whereas let-7 miRNA NL5901 silenced worms exhibited a thrashing count 51.80 ± 1.583 (*N* = 10), thus exhibiting 12.93% (*p* < 0.01) and 28.76% (*p* < 0.001) increased motility as compared to N2 and NL5901 strains (**Figure 6**).

### Knockdown of Let-7 miRNA Increases Oxidative Stress

**FIGURE 7:**
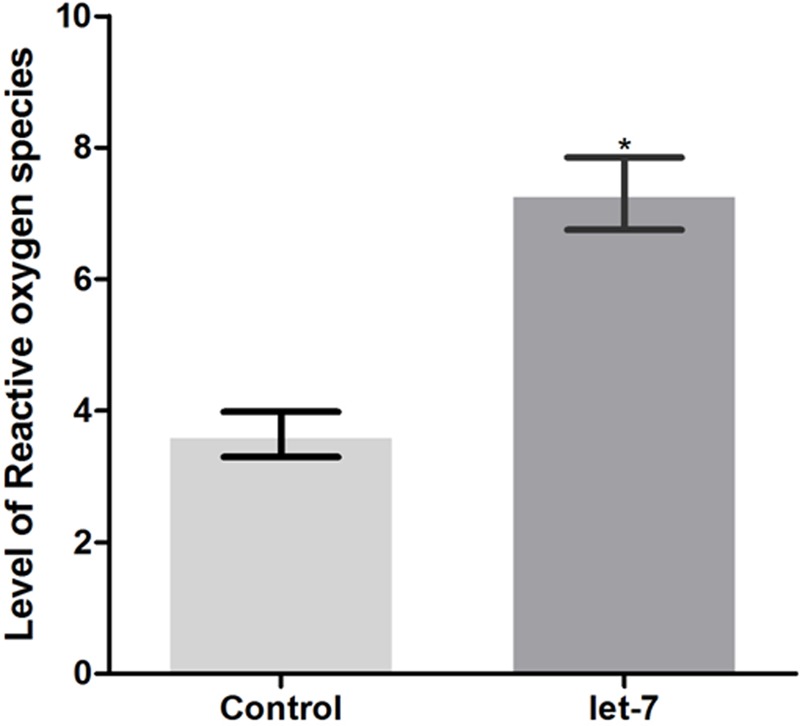
ROS production levels as estimated by *H_2_DCFDA* assay in wild type strain N2 (control and let-7 knockdown condition). ^∗^*p* < 0.05.

Neurodegenerative PD is characterized by mitochondrial dysfunction. Altered mitochondrial function is implicated in increased reactive oxygen species (ROS) and cellular energy impairment (Guo et al., 2013). We studied the effect of let-7 miRNA knockdown on the alteration of ROS level. Employing H_2_DCFDA assay we checked ROS level in control and let-7 miRNA knockdown groups. We observed fluorescence intensity of 3.636 ± 0.3434 relative fluorescence intensity units (RFU) per worm in control group whereas let-7 miRNA knockdown worms exhibited fluorescence intensity of 7.300 ± 0.5500 RFU per worm, thereby displaying 50.19% (*p* < 0.05) increased ROS level with respect to that of control group (**Figure 7**).

### Let-7 miRNA Silenced Worms Displayed Enhanced Fat Content

**FIGURE 8:**
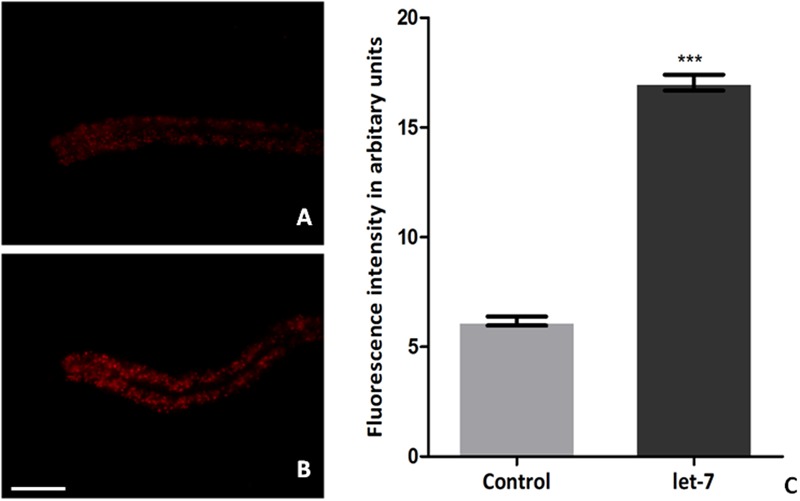
Nile red staining for fat content in *C. elegans* from control **(A)**, let-7 miRNA silenced group **(B)**, and graphical representation for fluorescence intensity of the worms as quantified using ImageJ software **(C)**. ^∗∗∗^*p* < 0.001. Scale bar: 50 μm.

Low level of fat deposition is known to associate with PD (Lorefalt et al., 2009; Bernhardt et al., 2016). In order to delineate the effect of let-7 miRNA in fat deposition, we employed Nile red for assaying the fat deposition in worms of control and let-7 miRNA silenced group. Fluorescent intensity of stained fat was analyzed through fluorescence microscopy and quantified using ImageJ software. We observed optimal level of fat content in worms of control group (**Figure 8A**) where the mean fluorescence intensity was 6.174 ± 0.2050 (*N* = 5) arbitrary units. Staining intensity in the worms of let-7 miRNA silenced group was increased (**Figure 8B**) and mean fluorescence intensity was 17.04 ± 0.3631 (*N* = 5), thereby exhibiting 2.75-fold increased (*p* < 0.001) fat content as compared to that of control group (**Figure 8C**).

### Let-7 miRNA Knockdown Had No Effect on Acetylcholinergic and Dopaminergic Neurons

**FIGURE 9:**
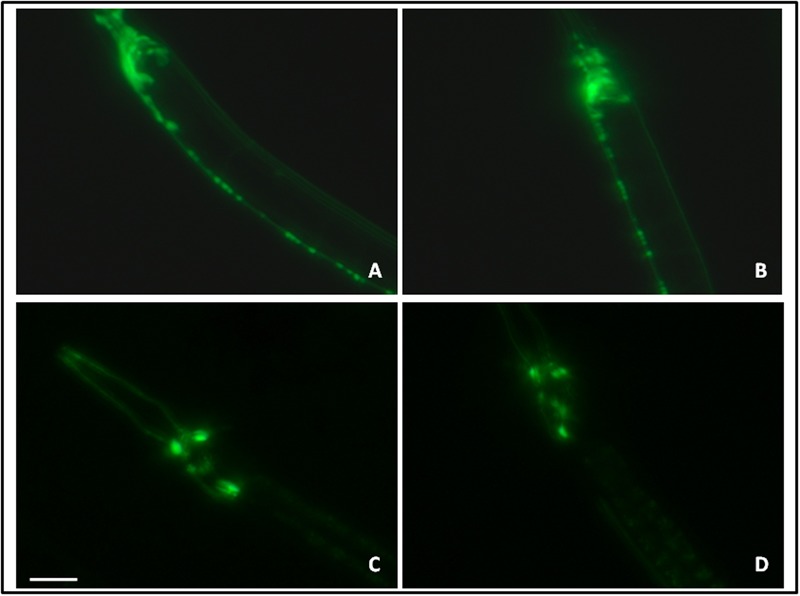
GFP expression pattern in the *unc-17*::GFP strain (**A**: control, **B**: lct-7 miRNA knockdown) and dat-l::GFP strain (**C**: control and **D**: let-7 miRNA knockdown) using fluorescence microscopy. Scale bar: 50 μm.

The effect of let-7 miRNA knockdown on acetylcholinergic and dopaminergic neurons was studied via expression of GFP tagged with *unc-17* and *dat-1* transporter of acetylcholinergic and dopaminergic neurons, respectively. Transgenic strains LX929 and BZ555 were used for this study and expression of the transgenes was studied via fluorescence microscopy. We observed no significant effect on expression of GFP either in let-7 miRNA knockdown LX929 or BZ555 strain as compared to their respective controls (**Figures 9A–D**), suggesting that knockdown of let-7 miRNA has no effect on these neuronal subpopulations.

### Dopamine Associated Function Is Not Affected under Let-7 miRNA Silencing

**FIGURE 10:**
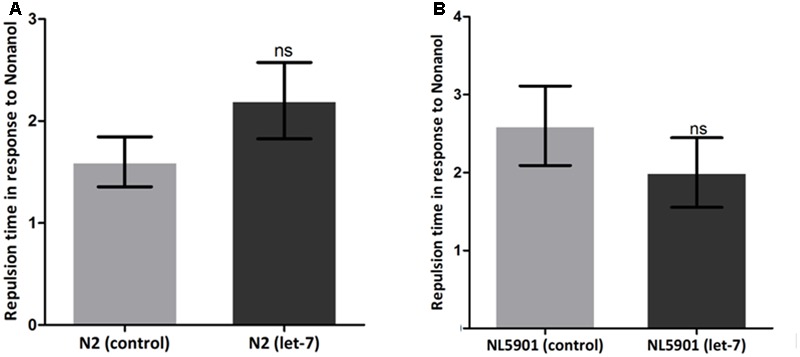
Estimation of dopamine content employing nonanol assay. *C. elegans* wild type strain N2 {control and let-7 knockdown} **(A)**. Transgenic strain NL5901 expressing human alpha-synuclein {control and lct-7 miRNA knockdown} **(B)**. ns, non-significant.

Various behavioral functions of worms are regulated by neurotransmitter dopamine (Sawin et al., 2000). Alterations in dopamine related functions are associated with PD (Li et al., 2015). To assess the effect of let-7 knockdown on dopamine function, we employed the odor-based repellent assay using 1-nonanol for various conditions. We observed that wild type N2 strain exhibited a mean response time of 1.600 ± 0.2449 s (*N* = 10) whereas the mean response time of let-7 miRNA knockdown worms was 2.200 ± 0.3742 s (*N* = 10) (**Figure 10A**). In contrast transgenic strain NL5901 displayed mean response time of 2.600 ± 0.5099 s (*N* = 10) and silencing of let-7 miRNA in this strain resulted in a mean response time of 2.000 ± 0.4472 s (*N* = 10) (**Figure 10B**). Worms under let-7 silenced condition did not exhibit any significant change in their functions associated with neurotransmitter dopamine as studied via nonanol repulsion assay.

## Discussion

The expression of miRNA varies for different tissues, and any deviation from its normal state may lead to various conditions including diseases like NDs (Tan et al., 2015; Basak et al., 2016). There are many findings that report on the importance of miRNAs in disease progression (Sempere et al., 2004). The miRNA regulatory event is very complex in nature with many intersecting pathways. miRNAs regulate transcriptional factors and in turn are being regulated by genes that code for RNA binding proteins. The regulatory network and feedback loop is used by miRNA for maintaining optimal level of gene expression pattern during body and organ development such as during the development of nervous system and cardiovascular system (Nam et al., 2011). Loss or over-expression of specific miRNA in elderly vertebrate animals results in neurodegeneration (Bicchi et al., 2013). Let-7 is an evolutionarily conserved miRNA that has been reported to repress multiple oncogenes by affecting key regulators of the cell cycle, cell differentiation, and apoptotic pathways. Let-7 miRNA is differentially expressed in alpha-synuclein transgenic animals and human Parkin ortholog *pdr-1* mutant animals (Asikainen et al., 2010). Let-7 miRNA are also downregulated by pathogenic LRRK2 (Gehrke et al., 2010). Let-7 miRNA, by bioinformatics analysis, is known to regulate genes of cell death, autophagy, mTOR and insulin pathway. *C. elegans* homolog of amyloid precursor protein *apl-1* is also reported to be controlled by let-7 miRNA (Yokota et al., 2003; Revuelta et al., 2008). We observed that let-7 miRNA was over expressed in *C. elegans* model of PD expressing wild type ‘human’ alpha-synuclein protein, while its expression was reduced in *C. elegans* model expressing mutant alpha-synuclein. Toward our studies of exploring the importance of let-7 miRNA in the context of PD, we constructed an RNAi feeding bacterial clone of let-7 miRNA and studied it employing transgenic *C. elegans* strain expressing human alpha-synuclein. Our results suggest that loss of function of let-7 miRNA results in significant upregulation of *daf-12* and *daf-16* gene expression validating the fact that let-7 miRNA controls the expression level of *daf-12* and *daf-16* mRNA. *daf-12* mRNA acts as a downstream target of let-7 miRNA as reported previously (Hammell et al., 2009). *daf-12* and *daf-16* encode member of the steroid hormone receptor superfamily and FOXO transcriptional factor, respectively, both reported to regulate the pathogenesis associated with PD (Haque and Nazir, 2014; Mahanti et al., 2014). In our studies, we observed that alpha-synuclein accumulation, and end points associated with PD were decreased in the absence of let-7 miRNA indicating the importance of let-7 miRNA directly with the progression of NDs. Over-expression of alpha-synuclein leads to degeneration of dopaminergic neurons (Cao et al., 2010). Therefore, silencing of let-7 miRNA might be protecting the dopaminergic neurons via decreasing the accumulation of alpha-synuclein. Further, since decrease in the accumulation of alpha-synuclein protein is associated with enhanced autophagy, we quantified mRNA levels of different autophagy marker genes. Our findings indicate that mRNA levels of *lgg-1* and *atg-13* were increased in let-7 silenced worms. GFP::LGG-1 was also increased in let-7 knockdown worms that further validate the previous findings. *lgg-1* is an ortholog of *Saccharomyces cerevisiae* Atg8p and mammalian MAP-LC3, which is required for degradation of cellular components, *atg-13* is an autophagy related gene required for autophagosome formation. Our studies suggest that the targets of let-7 miRNA might be involved in autophagy pathway which was increased in the absence of let-7 miRNA.

A defect in locomotion due to imbalance of neurotransmitters is the hallmark of PD. In order to understand the effect of let-7 miRNA knockdown on normal locomotory behavior we employed thrashing assay to quantify motility in the worms. Our studies showed enhanced motility which suggests let-7 miRNA may have role in excitatory neurotransmission. Further, we studied the effect of let-7 miRNA silencing on dopamine function via nonanol repulsion assay. Our results indicate no direct role of let-7 miRNA on functions associated with dopamine content. It suggests that loss of let-7 miRNA function does not have any effect on dopamine synthesis or overall availability. This can be explained by the fact that expression of some miRNAs is very specific and its alteration in expression leads to death of particular cell. Keeping this in mind we evaluated the effect of let-7 miRNA knockdown on dopaminergic and acetylcholinergic neurons. Our findings indicate that absence of let-7 miRNA has no effect on these neurons. This provides a clue toward protective role of let-7 miRNA in cell death. So, we next examined the effect of let-7 miRNA silencing on programmed cell death associated genes. Our studies indicate that knockdown of let-7 miRNA protects cells from death by reducing the *ced-4* and *jnk-1* mRNA expression. *ced-4* is an enzyme required for initiation of programmed cell death by activating *ced-3* which encodes a caspase essential for execution of apoptosis (Huang et al., 2013). *jnk-1* is the variant of Jun N-terminal kinases (JNKs), which belongs to MAP-kinase superfamily. JNK-1 mediates apoptotic signaling in both extrinsic and intrinsic pathways (Dhanasekaran and Reddy, 2008). Our finding indicates that loss of let-7 miRNA might play protective role in *C. elegans*.

Parkinson’s disease is known to be associated with oxidative stress. Hence, we studied ROS in the worms at the basal level and after silencing of let-7 miRNA. In our studies, we observed elevated level of ROS. Many research groups have reported that elevated ROS leads to induction of autophagy (Chen et al., 2009; Filomeni et al., 2015). Silencing of let-7 miRNA leads to elevated ROS level and mild increase in ROS level acts as inducer of autophagy pathway. This study provides evidence that any increase in the level of ROS leads to the increase in autophagy that might help in the clearance of misfolded protein aggregates. Mild increases in ROS level also promote the longevity by activating the *hif-1* which encodes hypoxia inducible factor (HIF-1), a highly conserved transcription factor that activates survival promoting genes during hypoxia (Lee et al., 2010). Thus absence of let-7 miRNA might help in the reduction of alpha-synuclein protein aggregates in *C. elegans* model and enhancing life span. Altered lipid content is known to be associated with PD (Lorefalt et al., 2009; Bernhardt et al., 2016). Reduced level of fat content has been seen in the worms expressing human alpha-synuclein protein. Previous study showed that *C. elegans* model of PD has low level of lipid content (Jadiya et al., 2011). Our study shows fat content was increased by decreasing the expression of alpha-synuclein in let-7 miRNA silenced worms. This suggests that absence of let-7 miRNA might help in maintaining lipid content in worms.

## Conclusion

Our study leads to an understanding of the role of *C. elegans* let-7 miRNA in progression of PD and confirms that absence of let-7 miRNA leads to decrease in accumulation of alpha-synuclein protein in transgenic worms. Our studies further provide a clue toward the role of let-7 miRNA in possibly decreasing alpha-synuclein expression via increasing autophagy and increasing *daf-16* FOXO transcription factor. Decrease in the expression of alpha-synuclein protein via Daf-16 has been reported (Pino et al., 2014). Our studies prove that loss of let-7 miRNA did not affect the dopaminergic and acetylcholinergic neurons. Our results also open avenues for further research toward deciphering the importance of let-7 miRNA in the context of various other diseases and may prove to be beneficial target for the treatment of PD in future. Let-7 could particularly be studied for its role in modulating the multi-factorial aspect of neurodegenerative PD.

## Author Contributions

Shamsuzzama and AN conceived the studies. Shamsuzzama and LK performed the experiments. Shamsuzzama and AN wrote the paper. AN provided reagents and guidance.

## Conflict of Interest Statement

The authors declare that the research was conducted in the absence of any commercial or financial relationships that could be construed as a potential conflict of interest.

## Footnotes

1 http://www.mirbase.org/

2 http://www.mirbase.org/
